# Comparison of pregnancy outcomes and placental characteristics between selective fetal growth restriction with and without thick arterio-arterial anastomosis in monochorionic diamniotic twins

**DOI:** 10.1186/s12884-021-04346-8

**Published:** 2022-01-06

**Authors:** Xueju Wang, Luyao Li, Pengbo Yuan, Yangyu Zhao, Yuan Wei

**Affiliations:** Department of Obstetrics and Gynecology, Peking University Third Hospital, No. 49 Hua Yuan North Road, Hai Dian District, Beijing, 100191 China

**Keywords:** Selective fetal growth restriction, Placenta, Thick arterio-arterial anastomoses, Monochorionic diamniotic twins

## Abstract

**Background:**

Unequal placental territory in monochorionic diamniotic twins is a primary cause of selective fetal growth restriction (sFGR), and vascular anastomoses play important role in determining sFGR prognosis. This study investigated differences in placental characteristics and pregnancy outcomes in cases of sFGR with and without thick arterio-arterial anastomosis (AAA).

**Methods:**

A total of 253 patients diagnosed with sFGR between April 2013 and April 2020 were retrospectively analyzed. An AAA greater than 2 mm in diameter was defined as a thick AAA. We compared placental characteristics and pregnancy outcomes between cases of sFGR with and without thick AAA.

**Results:**

Prevalence of AAA, thick arterio-venous anastomosis (AVA), veno-venous anastomosis (VVA), and thick VVA were significantly higher in the thick AAA group relative to the non-thick AAA group (100.0 vs. 78.5%, *P* < 0.001; 44.3 vs. 15.4%, *P* < 0.001; 27.1 vs. 10.8%, *P* = 0.017, and 24.3 vs. 6.2%, *P* = 0.004, respectively). The total numbers of AVA and total anastomoses were significantly higher in thick AAA group relative to the non-thick AAA group (5 [1, 14] vs. 3 [1, 15, *P* = 0.016; and 6 [1, 15] vs. 5 [1, 16], *P* = 0.022, respectively). The total diameter of AAA, AVA, VVA, and all anastomoses in the thick AAA group was larger than in the non-thick AAA group (3.4 [2.0,7.1] vs. 1.4 [0.0, 3.3], *P* < 0.001; 6.3 [0.3, 12.0] vs. 2.5 [0.3, 17.8], *P* < 0.001; 4.2±1.8 vs. 1.9±1.2, *P* =0.004; and 10.7 [3.2,22.4] vs. 4.4 [0.5, 19.3], *P* < 0.001, respectively). Growth-restricted fetuses in the thick AAA group exhibited significantly increased birthweight relative to those in thenon-thick AAA group (1570 (530, 2460)g vs. 1230 (610, 2480)g, *p* = 0.002).

**Conclusions:**

In the placentas associated with sFGR, thick AAA can co-occur with thick AVA and VVA, and placental angiogenesis may differ significantly based upon whether or not thick AAA is present. The birth weights of growth-restricted fetuses in cases of sFGR with thick AAA are larger than in cases without thick AAA.

## Background

Selective fetal growth restriction (sFGR) is a complication that is unique to monochorionic diamniotic (MCDA) twin pregnancies, affecting an estimated 10-15% of MCDA twins [1, 2]. The primary causes of sFGR are believed to include unequal placental sharing and abnormal umbilical cord insertion in the context of MCDA twins sharing the same placenta, whereas placental superficial vascular anastomosis can profoundly influence the prognosis and progression of sFGR [1, 3, 4].

Arterio-arterial anastomosis (AAA) permits rapid bidirectional blood flow, leading to the equalization of hemodynamic pressures in the two MCDA fetuses to some degree [4]. Zhao et al. found that superficial AAA was present in as many as 98% of cases of sFGR, with diameters of affected vessels being significantly larger than those observed in normal MCDA twins [5]. An AAA greater than 2 mm in diameter is defined as a thick AAA [6, 7]. Gratacos et al. found that thick AAA was significantly more common in cases of type III sFGR relative to types I and II, with these anastomoses being related to the end-diastolic velocity of the fetal umbilical arteries [6]. Whether thick AAA impacts sFGR-related pregnancy outcomes, however, remains to be firmly established, and there have been few studies to date regarding differences in placental characteristics between cases of sFGR with and without thick AAA. The present study was therefore designed to compare pregnancy outcomes and placental characteristics between cases of sFGR with and without thick AAA, and to understand how thick AAA influences sFGR.

## Methods

For this study, patients with MCDA twins exhibiting sFGR that had been admitted to the Department of Obstetrics in the Peking University Third Hospital between April 2013 and April 2020 were retrospectively analyzed. Each monochorionic placenta diagnosed prenatally via ultrasound was pathologically examined after delivery and subjected to dye injection with the approval of the Ethics Committee of Peking University Third Hospital and the informed consent of patients. Patients were excluded from this study if they had been diagnosed with twin anemia-polycythemia sequence (TAPS) or twin-to-twin transfusion syndrome (TTTS), had undergone fetal reduction, experienced fetal demise during pregnancy, or exhibited placental rupture following delivery. Those patients that remained were separated into two groups based upon whether or not thick AAA was present in the placenta. Placental characteristics and pregnancy outcomes were then compared in these two groups.

sFGR in MCDA twins was defined based upon the detection of an ultrasound-based weight estimate below the 10^th^ percentile for the corresponding gestational age for either fetus [2]. The criteria previously published by Gratacos et al. were used to guide sFGR classification [6]. Type I sFGR presents with normal fetal umbilical artery end-diastolic blood flow, while in type II sFGR these arteries exhibit continuously absent or reversed end-diastolic blood flow, and in type III sFGR the end-diastolic velocity of these arteries is intermittently absent or reversed. The management of sFGR was based on the twin pregnancy guidelines issued by China in 2015, as well as parents' wishes and NICU treatment conditions. We recorded the final sFGR classification associated with a given pregnancy prior to delivery, given that these classifications can change during pregnancy [8]. TTTS was diagnosed according to the combined presence of a maximum vertical pocket (MVP) ≥ 8 cm in one sac and ≤ 2 cm in the other sac irrespective of gestational age at diagnosis [9]. Antenatal TAPS was diagnosed based upon discordant measurements of the middle cerebral artery peak systolic velocity (MCA-PSV) being > 1.5 the multiple of the median (MoM) in donors, and < 1.0 in recipients [10]. Postnatal TAPS was diagnosed based upon inter-twin hemoglobin differences > 8 g/dL together with reticulocyte count ratio >1.7 and/or placental anastomoses less than 1 mm detected upon dye injection [10]. Delivery mode, postnatal Apgar scores, birth weight, and umbilical artery pH were recorded. In China, neonatal asphyxia is diagnosed based upon a 1-minute Apgar score < 7 together with an umbilical artery pH < 7.2 [11]. Birthweight discordance ratio values were calculated as follows: (birthweight_heavier fetus_ – birthweight_lighter fetus_)/birthweight_heavier fetus_.

**Fig. 1:**
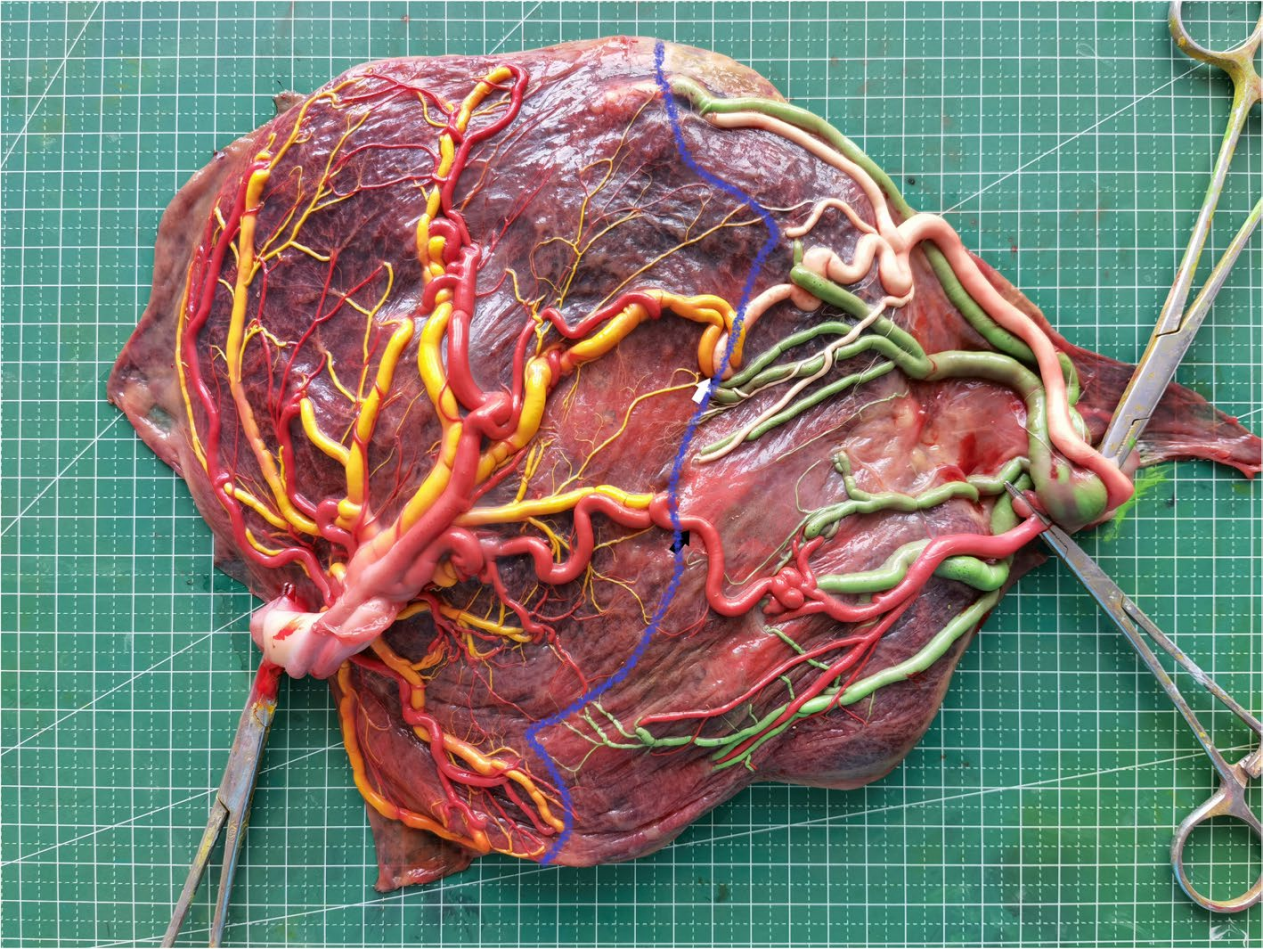
A representative placenta following dye injection (black arrow, AAA arterio-arterial anastomosis; white arrow, AVA arterio-venous anastomosis; blue curve, vascular equator)

Placental dye injection was conducted as per a protocol previously published by our center [7, 12–14], with a representative high-resolution image of an injected placenta being shown in Fig. 1.

All placental images were analyzed using ImageJ (v. 1.51j8, NIH, MD, USA). Superficial AAA and veno-venous anastomosis (VVA) were detected based upon direct external communication between pairs of homonymous umbilical vessels, with the caliber of these anastomoses being measured as the minimum external diameter along the course of the anastomosis. Deep arterio-venous anastomoses (AVA) were defined as sites where an unpaired artery from one twin had penetrated the chorionic plate within less than 1.0 mm of an unpaired vein from the other twin. AVA diameters were defined by the narrowest diameter of the feeding artery. A given anastomosis was defined as thick when its diameter was ≥2 mm [6, 7]. The maximum placental diameter was defined as the maximal distance between the placental parenchyma margins. The distance between two umbilical cord insertion points was measured based upon the center of each insertion point. For velamentous umbilical cords, the margin of the adjacent placental parenchyma was selected as the center of the insertion point [12]. Marginal umbilical insertion included both velamentous insertion and a distance < 1 cm between insertion points to the margins of the placental parenchyma [4].

Placental territories corresponding to each fetus were defined based upon the margins for injected dyes, with each territory being expressed as a fraction of the overall placental area. Measurements of placental territory area values were made using ImageJ. The vascular equator corresponded to the boundary between these two placental territories, and was first defined based upon the locations of all anastomoses. The placental territory corresponding to each twin was then measured with a freehand selection tool in the ImageJ software (Fig. 1).

All statistical analyses were conducted using SPSS 24.0. Data are given as means ± standard deviation or median (maximum, minimum) for normally and non-normally distributed data, respectively. Quantitative data that were and were not normally distributed were analyzed via Student’s t-tests and Mann-Whitney U tests, respectively. Enumerated data were compared via Chi-squared tests or Fisher’s exact test when ≥ 40 and < 40 samples were analyzed, respectively. P < 0.05 was the significance threshold.

## Results

**Fig. 2:**
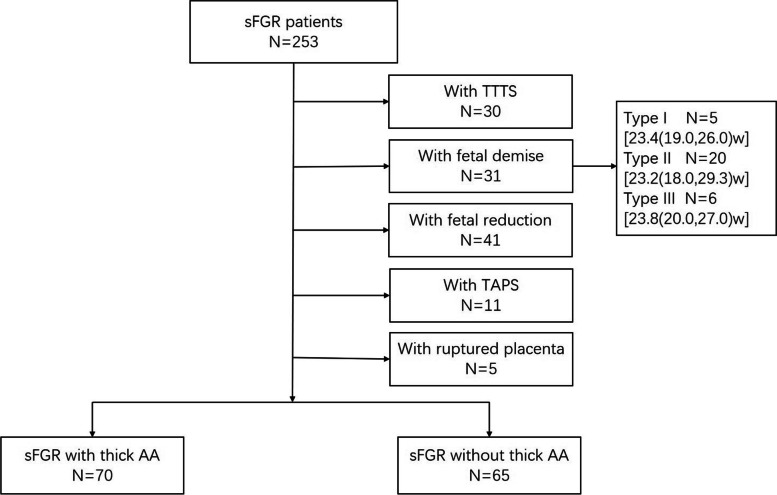
Patient inclusion criteria flowchart

In total, 253 patients were diagnosed with sFGR of MCDA twins and admitted to the Department of Obstetrics in the Peking University Third Hospital between April 2013 and April 2020 and were eligible for inclusion in the present study (Fig. 2).

Information pertaining to general conditions and pregnancy outcomes in these two groups is compiled in Table 1.

**Table 1:** Comparison of baseline and pregnancy outcomes of sFGR with and without thick AAA

	Thick AAA group (*n* =70)	Non-thick AAA group (*n* =65)	*P*-value
Age (year)	28(20,42)	30(20,42)	0.218
Pregnancy induced hypertension [cases (%)]	11(15.7)	16(24.6)	0.282
Gestational diabetes mellitus [cases (%)]	15(21.4)	12(18.5)	0.830
Gestational age at diagnosis (weeks)	25.7±4.9	25.7±5.0	0.999
Final classification			
sFGR type I [cases (%)]	32(45.7)	38 (58.5)	0.011
sFGR type II [cases (%)]	17(24.3)	21 (32.3)	
sFGR type III [cases (%)]	21(30.0)	6 (9.2)	
Gestational age at delivery (weeks)	34.0±2.5	33.2±2.4	0.057
Delivery mode			
Vaginal birth [cases (%)]	4(5.7)	9(13.8)	0.147
Cesarean section [cases (%)]	66(94.3)	56(86.2)	
Birthweight of normally growing fetuses (g)	2160±492	2016±483	0.088
Number of neonatal asphyxias [cases (%)]	6(8.6)	6(9.2)	1.000
Birthweight of growth-restricted fetuses (g)	1570(530,2460)	1230(610,2480)	0.002
Number of neonatal asphyxias [cases (%)]	5(7.1)	6(9.2)	0.758
Birthweight discordance ratio	0.53±0.21	0.48±0.18	0.123

*sFGR* selective fetal growth restriction, *AAA* arterio-arterial anastomosis

The gestational age at diagnosis in the thick AAA group was significantly higher than in the non-thick AAA group. Type III sFGR rates in the thick AAA group were significantly higher than in the non-thick AAA group. Growth-restricted fetuses in the thick AAA group exhibited significantly increased birthweight relative to those in the non-thick AAA group.

The placental characteristics in these two groups are compared in Table 2. The prevalence of AAA, AVA, VVA, and thick VVA was significantly higher in the thick AAA group relative to the non-thick AAA group. The total number of AVA and all anastomoses in the thick AAA group was significantly larger than that in the non-thick AAA group. The total diameters of AAA, AVA, VVA, and all anastomoses in the thick AAA group were significantly greater than in the non-thick AAA group.

**Table 2:** Comparisons of placental characteristics in cases of sFGR with and without thick AAA

	Thick AA group (*n* =70)	Non-thick AA group (*n* =65)	*P*-value
Prevalence of AAA [cases (%)]	70(100.0)	51(78.5)	<0.001
Prevalence of thick AAA [cases (%)]	70(100.0)	0	<0.001
Total number of AAA(n)	1(1,3)	1(1,2)	0.125
Total diameter of AAA (mm)	3.4(2.0,7.1)	1.4(0.0,3.3)	<0.001
Prevalence of AVA [cases (%)]	65(92.9)	62(95.4)	0.720
Prevalence of thick AVA [cases (%)]	31(44.3)	10(15.4)	<0.001
Total number of AVA(n)	5(1,14)	3(1,15)	0.016
Total diameter of AVA (mm)	6.3(0.3,12.0)	2.5(0.3,17.8)	<0.001
Prevalence of VVA [cases (%)]	19(27.1)	7(10.8)	0.017
Prevalence of thick VVA [cases (%)]	17(24.3)	4(6.2)	0.004
Total number of VVA(n)	1(1, 2)	1(1, 2)	0.821
Total diameter of VVA (mm)	4.2±1.8	1.9±1.2	0.004
Total number of all the anastomoses(n)	6(1, 15)	5(1, 16)	0.022
Total diameter of all the anastomoses (mm)	10.7(3.2,22.4)	4.4(0.5,19.3)	<0.001
Placental territory discordance ratio	0.26±0.09	0.34±0.15	0.001
Marginal umbilical insertion of normal growth fetuses[cases (%)]	6(8.6)	7(10.8)	0.774
Marginal umbilical insertion of restricted growth fetuses[cases (%)]	56(80.0)	55(84.6)	0.509
Birthweight discordance / Placental territory discordance	0.48(0.15,24.07)	0.73(0.13,3.71)	0.001
Umbilical insertion ratio	0.58(0.09,1.00)	0.61(0.08,1.00)	0.167

*sFGR* selective fetal growth restriction, *AAA* arterio-arterial anastomosis, *AVA* arterio-venous anastomosis, *VVA* veno-venous anastomosis

Both the placental territory discordance ratio and the ratio of birthweight discordance to placental territory discordance were lower in the thick AAA group relative to the non-thick AAA group.

## Discussion

The incidence of sFGR in MCDA twins is associated with unique placental angioarchitectural features [3, 5, 15]. In the present analyses, we were able to expand on these prior findings by demonstrating that the placental angioarchitecture differed significantly between cases of sFGR with and without thick AAA, indicating that placental angiogenesis may differ between these groups. We observed significantly higher rates of both thick AVA and thick VVA in the thick AAA group relative to the non-thick AAA group, with the total diameters of AAA, AVA, VVA, and total anastomoses in the thick AAA group being significantly larger than in the non-thick AAA group. These findings indicate that these three types of placental vascular anastomoses are thicker in the context of sFGR presenting with thick AAA relative to sFGR without thick AAA. These findings are consistent with those of Nikkels et al., who found AAA and VVA to always co-occur in MCDA twins [4]. We thus speculate that thick AAA can similarly co-occur with thick AVA and VVA in the context of sFGR.

Previous studies suggest that AAA permits bidirectional blood flow and can thereby equilibrate inter-twin blood volumes, thus protecting growth-restricted fetuses [4, 5, 16]. Consistent with such a model, we observed significantly higher birth weights for growth-restricted fetuses in the thick AAA group relative to the non-thick AAA group. However, we also observed a lower placental territory discordance ratio in the thick AAA group relative to the non-thick AAA group. Given that placental territory has the potential to impact birthweight, we calculated the ratio of birthweight discordance to placental territory discordance [17]. When this ratio is less than 1, this indicates that the degree of birthweight discordance is less than the degree of placental territory discordance such that this ratio could reflect the influence of vascular anastomoses on birthweight [17]. We found that the ratio in the thick AAA group was decreased relative to the non-thick AAA group, supporting the potential for thick AAA to protect growth-restricted fetuses with more compensatory effect by better permitting the equilibration of inter-twin blood volumes.

The present study revealed that the gestational age at diagnosis in the thick AAA group was significantly larger than in the non-thick AAA group, which means that the onset time of sFGR in the thick AAA group may be later than in the non-thick AAA group. As previously mentioned, thick AAA can protect the growth-restricted fetus to a greater extent by equilibrating more inter-twin blood volume, so we speculate that the onset time of sFGR with thick AAA can thus be delayed.

We also detected significant differences in sFGR types when comparing these two groups. Specifically, pairwise comparisons revealed that type III sFGR rates in the thick AAA group were significantly higher than the non-thick AAA group, consistent with previous studies [6, 15, 18]. Placentas in cases of type III sFGR exhibit the highest prevalence of thick AAA, which has the potential to profoundly alter inter-twin hemodynamics resulting in the intermittently absent or reversed end-diastolic velocity characteristics of the fetal umbilical arteries.

Based on the preliminary conclusions of this study, we posit that prior to fetoscopic laser surgery treatment of sFGR in order to reduce the risk of unexpected single fetal demise, individualized management should be considered for differences in placental characteristics in cases of sFGR with type III or thick AAA, as the laser coagulation of thicker anastomoses could be more challenging and lead to a higher percentage of chorionic damage for both fetuses.

There are certain limitations to the present analysis. Given the requirements associated with placental dye injection, only patients with two live fetuses and evidence of sFGR that underwent conservative treatment were eligible for inclusion in this study. Patients that experienced single fetal death or underwent fetal reduction were excluded from this analysis, potentially biasing results, particularly those pertaining to pregnancy outcome comparisons. We postulate that placental structure can impact pregnancy outcomes, and further studies of whether thick AAA have any relationship with the incidence of single or co-twin fetal death are thus warranted. Sun et al. were able to detect placental vascular anastomoses in MCDA twins using a combination of three-dimensional sonography and tomographic ultrasound imaging [19]. These non-invasive approaches offer promise as tools for the study of placental vascular anastomoses and the relationship between such anastomoses and pregnancy outcomes. Another limitation is the lack of AVA directionality as it often appears in the form of a vascular net, which makes it difficult to clarify its specific directionality and influence on hemodynamics. As the primary objective of this study was the comparison of placental characteristics between cases of sFGR with or without thick AAA, the lack of information regarding AVA directionality should have little influence on the conclusions of the study.

## Conclusion

In conclusion, the results of this study suggest that thick AAA can co-occur with thick AVA and VVA in cases of sFGR, and that there may be differences in placental angiogenesis between cases of sFGR with and without thick AAA. Thick AAA could be beneficial to the growth of growth-restricted fetuses.

## Data Availability

All placental data and patient information are available on request to the corresponding author.

## Abbreviations

sFGR: Selective fetal growth restriction
AAA: Arterio-arterial anastomosis
AVA: Arterio-venous anastomosis
VVA: Veno-venous anastomosis
MCDA: Monochorionic diamniotic
TAPS: Twin anemia polythemia sequence
TTTS: Twin-to-twin transfusion syndrome
MVP: Maximum vertical pocket
MCA-PSV: Middle cerebral artery peak systolic velocity
